# Recent advances in single-cell engineered live biotherapeutic products research for skin repair and disease treatment

**DOI:** 10.1038/s41522-023-00463-8

**Published:** 2023-12-08

**Authors:** Muhamad Aidilfitri Mohamad Roslan, Mohd Norfikri Omar, Nur Azlina Mohd Sharif, Nurul Hanun Ahmad Raston, Mohd Hafiz Arzmi, Hui-Min Neoh, Ahmad Bazli Ramzi

**Affiliations:** 1 Institute of Systems Biology (INBIOSIS), Universiti Kebangsaan Malaysia, 43600 UKM Bangi, Selangor Malaysia; 2 Department of Biological Sciences and Biotechnology, Faculty of Science and Technology, Universiti Kebangsaan Malaysia, 43600 UKM Bangi, Selangor Malaysia; 3 Department of Fundamental Dental & Medical Sciences, Kulliyyah of Dentistry, International Islamic University Malaysia, 25200 Kuantan, Pahang Malaysia; 4 Melbourne Dental School, The University of Melbourne, 3053 Melbourne, Victoria Australia; 5 UKM Medical Molecular Biology Institute (UMBI), Universiti Kebangsaan Malaysia, 56000 Cheras, Kuala Lumpur Malaysia

**Keywords:** Applied microbiology, Antimicrobials

## Abstract

The human microbiome has emerged as a key player in maintaining skin health, and dysbiosis has been linked to various skin disorders. Amidst growing concerns regarding the side effects of antibiotic treatments, the potential of live biotherapeutic products (LBPs) in restoring a healthy microbiome has garnered significant attention. This review aims to evaluate the current state of the art of the genetically or metabolically engineered LBPs, termed single-cell engineered LBPs (eLBPs), for skin repair and disease treatment. While some studies demonstrate promising outcomes, the translation of eLBPs into clinical applications remains a significant hurdle. Substantial concerns arise regarding the practical implementation and scalability of eLBPs, despite the evident potential they hold in targeting specific cells and delivering therapeutic agents. This review underscores the need for further research, robust clinical trials, and the exploration of current advances in eLBP-based bioengineered bacterial chassis and new outlooks to substantiate the viability and effectiveness of eLBPs as a transformative approach in skin repair and disease intervention.

## Introduction

The human microbiome is a highly complex system that plays a crucial role in maintaining human health such as facilitating digestion, regulating the immune system, and synthesizing essential vitamins and nutrients^1,2^. Dysbiosis, an imbalance in the microbiome in many body parts, has been linked to various pathological conditions, including skin disorders such as acne vulgaris, psoriasis, and atopic dermatitis (AD)^3–5^. Antibiotic treatments for skin conditions have resulted in detrimental side effects such as antibiotic resistance, quorum cheater development, commensals depletion, and recurrent infections^6–8^. Live biotherapeutic products (LBPs) offer a potential solution by reestablishing the equilibrium of a healthy microbiome and enhancing overall health, including the skin^9–14^.

Single-cell engineered LBPs (eLBPs) are genetically and/or metabolically engineered microorganisms that provide targeted therapeutics directly at the disease site^9^. The microbial engineering strategy primarily uses bacteria as synthetic biology and bioengineered chassis has shown promise in treating a range of skin conditions, including wound healing, skin regeneration, and cancer treatment^15,16^. Despite increasing interest in microbiome engineering, previous reviews have mostly focused on microbial living materials, and only a few have considered the use of whole intact cells^2,9,17–19^. A wide array of LBPs and probiotics for the skin have also been thoroughly reviewed, but the majority barely discussed eLBPs concerning skin therapy. The present review focuses on the current strategies and advancements in the development of eLBP-based treatments for skin repair and disease through evidence obtained from clinical and preclinical testing over the past 10 years (2014-2023), providing a comprehensive overview of the current state of the art of eLBP research for skin therapy and its future outlook.

### Skin microbiome

The human skin is a complex system that comprises a rich variety of microorganisms known as the skin microbiome. In general, skin microenvironments which comprised moist, dry, and sebaceous sites harbor distinct microbial ecosystems, yet exhibit similarities in terms of the species present. Sebaceous sites are primarily dominated by *Cutibacterium* and *Staphylococci*, while moist sites are predominantly inhabited by *Corynebacterium* and *Staphylococcus* species^20^. Dry skin areas contain comparatively lower numbers of bacteria, but exhibit a more diverse composition, including a wide array of Proteobacteria, in addition to skin commensal species^21^. The resilience of the deeper layers of the core skin microbiome is influenced by genetics, diet, and personal hygiene routines. Despite exposure to cleaning and cosmetic products, the surface microbial community remains relatively stable^22,23^. Applying lotions on dry skin improves hydration and skin components but does not significantly change the composition of commensal microorganisms such as *C. acnes*, *S. epidermidis*, and *S. hominis*^24^. Dysbiosis of the skin microbiome is topographically specific and is commonly associated with pathological conditions or diseases as shown in Supplementary Table 1^3,25–27^.

This review will highlight instances where natural LBPs have been utilized as skin disease intervention and subsequently introduce the concept of eLBPs and discuss recent preclinical and clinical trials involving eLBPs. Considering the current advancement of synthetic biology and biological engineering especially in bacterial strain modification, this review delves deeper into the recent development of non-commensal bioengineered bacteria towards expanding the repertoire of bacterial chassis tailored for desired biological and biotherapeutic applications.

### Skin disease intervention using live biotherapeutic products

**Fig. 1:**
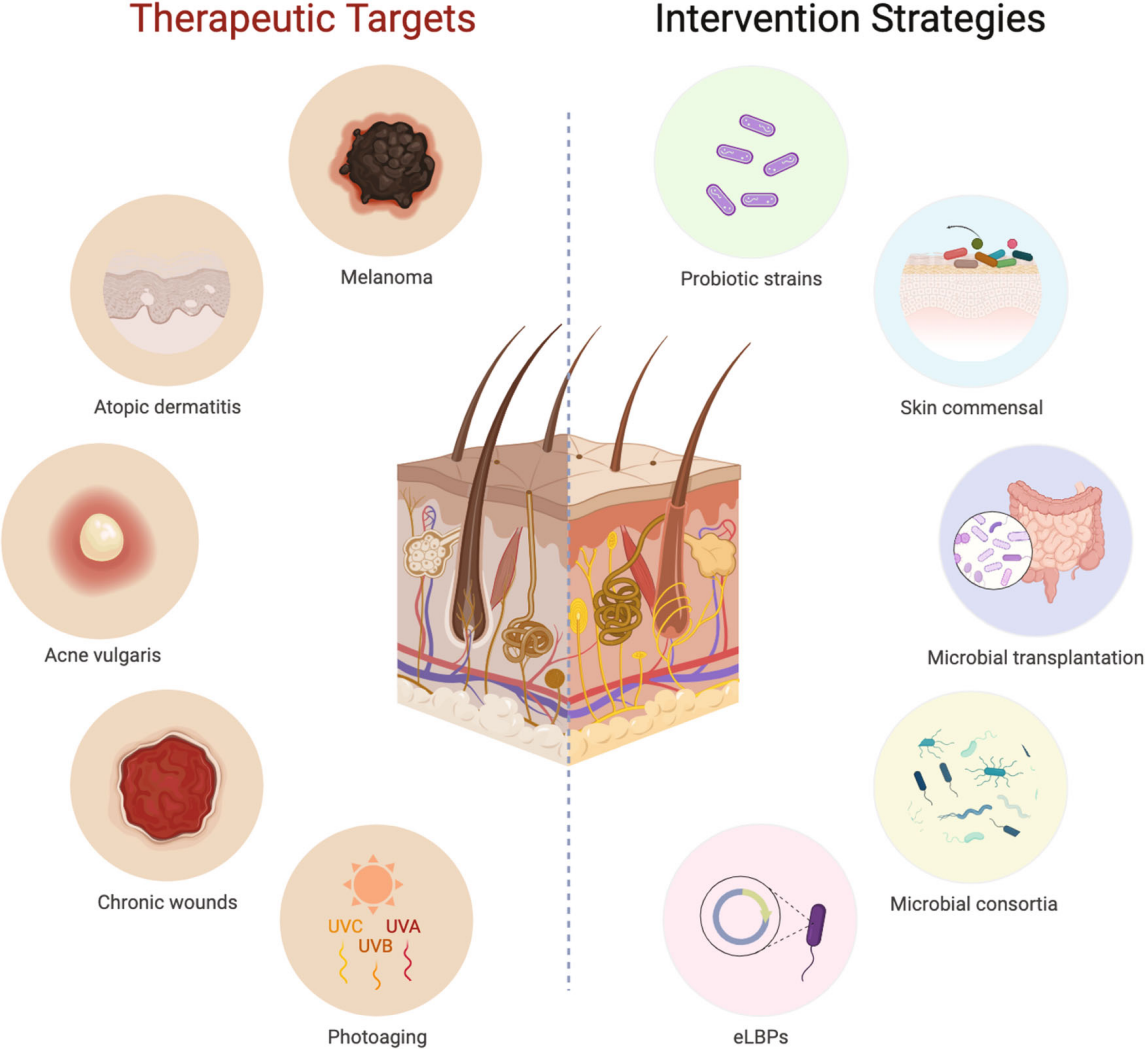
Intervention strategies through microbiome engineering for addressing prevalent skin conditions and diseases (therapeutic targets) using live biotherapeutic products.

One prevalent strategy to modulate the composition and/or function of the human microbiome (Fig. 1) involves the use of probiotics^28–30^. There is a growing body of evidence to suggest that probiotics can effectively impact the composition and metabolic activities of the human microbiome including the skin^12–14,31^. Conventional oral probiotic treatments have been proven to significantly reduce the severity of AD, particularly in adults, by promoting immune response and gut impermeability^32^. Yet, evolution in current probiotics revealed that topically applied probiotics are equally effective in improving anti-oxidation and lowering inflammation, apart from regulating other age-related conditions through the equilibration of commensal microbes and alteration of functional metabolisms^33,34^.

Beneficial skin commensals have a prospective biotherapeutic role, particularly for the repair and differentiation of the epidermal barrier^35^. For instance, certain strains of commensal *S. epidermidis* can express serine protease glutamyl endopeptidase and β-defensins, as well as activate Gamma delta T cells and upregulate Perforin-2, all of which inhibit the formation of pathogenic *S. aureus* biofilms in AD^36–39^, and induce interleukin (IL)-8 and neutrophils to combat inflammation in acne^40,41^. Additionally, scientists discovered that *S. epidermidis* (strain MO34 and MO38), which produces 6-N-hydroxyaminopurine (6-HAP), a molecule that hinders DNA polymerase activity, holds promise for providing defense against neoplasia. This breakthrough has unveiled a fresh perspective on the role of skin commensals in host protection against cancer^42^. In another case, the clinical trial reports of AD patients treated with the commensal *Roseomonas mucosa* showed better skin epithelial barrier function and decreased *S. aureus* load due to glycerophospholipids synthesis, which activated tissue-repair pathways^43,44^.

Skin commensals offer the potential for a more robust skin microbiome engineering, such as skin microbiome transplantation (SMT) to treat dysbiosis. SMT involves transplanting healthy skin microbiomes to the dysbiotic area^45^. A proof-of-concept study showed that unidirectional SMT, which transferred DNA markers partially from the forearm to the back of the same individual, was feasible^46^. As a result, SMT is being proposed as a solution to address underarm odour by replacing odour-causing commensals with new commensals obtained from a non-odorous donour^47^. Notably, ongoing research on SMT remains in its early phases, and there is yet to be an established, standardized procedure for its implementation. Contrary to SMT, fecal microbiome transplantation (FMT) stands as a well-established procedure and has shown promise in treating skin diseases, particularly through its influence on the gut-skin axis^48–50^. A recent clinical efficacy report of FMT on AD patients revealed a significant reduction in the average Scoring Atopic Dermatitis Score (SCORAD) from baseline, with a remarkable 75% decrease observed after just four treatments^50^.

Besides single-cell bacteriotherapy and microbiome transplantation, there is growing evidence that specific groups of microorganisms, when isolated and enriched, can manipulate the physiological functions of the host. For instance, an 11-member commensal consortium extracted from the fecal matter of healthy donors has been shown to stimulate the production of interferon-γ-producing CD8 T cells in the intestine which completely ablate and inhibit the metastasis of adenocarcinoma and melanoma cells^51^. Another *Lactobacilli* consortium showed promise in reducing inflammatory lesions by reducing the abundance of *Staphylococci* and *C. acnes* in a placebo-controlled study^34^. While microbial consortia may have greater impacts in manipulating host physiological functions, single-cell LBPs are relatively simpler to monitor and exploit, therefore are more practical for treating cutaneous diseases. Examples of LBPs formulated as oral and topical applications to treat skin conditions and diseases are presented in Supplementary Table 2.

**Fig. 2:**
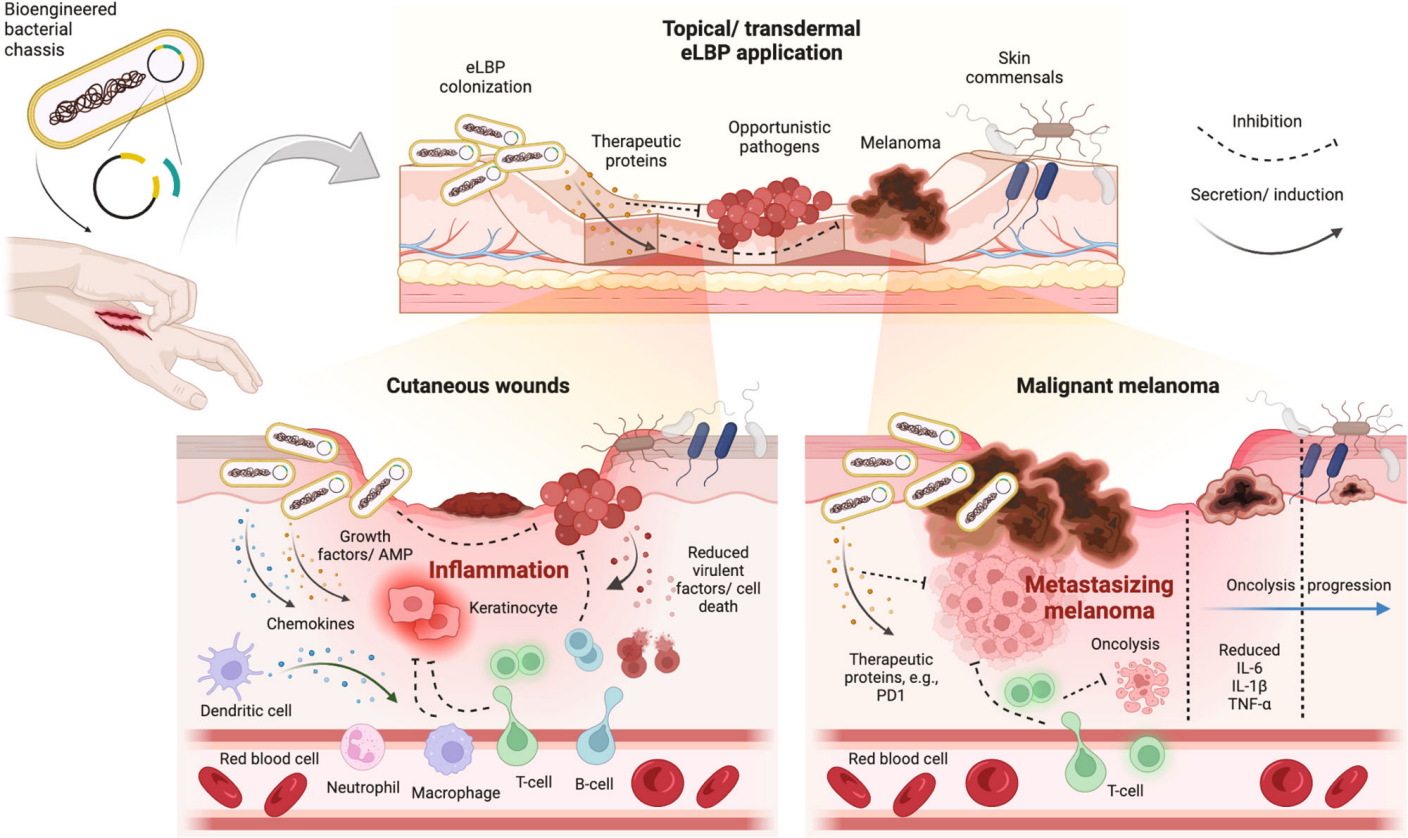
Schematic illustration and therapeutic strategy of engineered live biotherapeutic products (eLBPs) in treating skin conditions and dysbiosis such as cutaneous wounds and malignant melanoma.

Single-cell eLBPs are a cutting-edge field of research that utilizes genetically or metabolically engineered live microorganisms to perform specific functions, such as producing therapeutic compounds or targeting specific pathogen^9^. eLBPs have the potential to revolutionize the way we approach skin repair and disease treatment, as they can be tailored to target specific sites, cells and pathways in the body^17,18^. There is limited information available regarding the use of eLBPs for skin treatment, and most studies have only tested their efficacy using in vitro or in vivo models (Table 1). This review primarily focuses on two leading areas of eLBP research: 1) cutaneous wound treatments, and 2) malignant melanoma therapeutics (Fig. 2).

**Table 1:** Recent advancement in eLBP preclinical and clinical trials.

Indication	Chassis	Strategy	Route	Phase	Reference
Papulopustular rosacea	*Staphylococcus epidermidis* ATR-04	Induce IL-8 secretion	Topical	Clinical phase 1b	NCT04731259, Azitra
Netherton syndrome	*Staphylococcus epidermidis* ATR-12	Produce serine protease inhibitor Lympho-epithelial Kazal-type related inhibitor	Topical	Clinical phase 1b	Azitra, Identifier number pending
Chronic wounds: diabetic foot ulcer	*Limosilactobacillus reuteri* (formerly known as *L*. *reuteri*)	Express CXCL12, reduce chemokine degradation	Topical	Clinical phase 1/2	NCT05608187, Ilya Pharma^53^
Cutaneous wounds	*Lactococcus lactis*	Express CXCL12 in tandem with yellow light-emitting diodes therapy	Topical	Preclinical	^54^
Cutaneous wounds	*Lactococcus lactis*	Hydrogel for wound dressing containing *Synechococcus elongatus* and CXCL12-expressing *L. lactis*	Topical	Preclinical	^56^
Cutaneous/ Diabetic wounds	*Lactococcus lactis*	Produce vascular endothelial growth factors	Topical	Preclinical	^59^
Cutaneous wounds	Ovoid magnetotactic MO-1	Eradicate *S. aureus* via magnetic hyperthermia	Topical	Preclinical	^63^
Melanoma	*Clostridium butyricum*	Ablate tumor in hypoxia region through conjugated TPApy photosensitizer	Intratumoral	Preclinical	^65^
Melanoma	*Salmonella typhimurium*	Interferon-gamma–induced therapy	Intradermal	Preclinical	^76^
Melanoma	*Salmonella typhimurium*	Express radiation-sensitizing inhibin alpha gene	Intradermal	Preclinical	^77^
Melanoma	*Salmonella typhimurium*	PD-1 knockdown with siRNA and pimozide drug therapy	Intradermal	Preclinical	^78^
Melanoma	*Staphylococcus epidermidis*	Elicits tumor-specific T cells through ovalbumin peptides expression	Intradermal & Topical	Preclinical	^73^
Melanoma	*Escherichia coli*	Secret HSulf-1 within, and anchor DOX-linked GLY nanoparticles on the surface	Intradermal	Preclinical	^67^
Melanoma	*Escherichia coli*	Engineered OMV for TRAIL-induced apoptosis	Intradermal	Preclinical	^16^

### Cutaneous wound treatments

In wound healing, the skin undergoes overlapping phases of hemostasis, inflammation, proliferation, and remodeling. In the inflammation stage, immune cells gather at the site of injury due to distress signals, cytokines, and chemokines produced by damaged cells^15^. Chemokines like CXCL12, have been shown to have beneficial effects in healing cutaneous wounds and bind to CXCR4 receptors on immune cells and keratinocytes. To translate this theoretical framework, Ilya Pharma developed a first-in-class drug candidate, which is a recombinant CXCL12-expressing *Lactobacillus reuteri* R2LC (ILP100)^15,52^. Experiments on mice and minipigs in both healthy and hyperglycemic conditions showed faster wound healing due to increased CXCL12 availability and increased TGF-β expression in macrophages, therefore hastening the formation of granulation tissue and thin epithelial layers. In the Phase 1 clinical trial, ILP100 shortened the time to initial healing among patients by an average of 6 days and by 10 days at the highest dose^53^. It additionally elevated the density of CXCL12+ cells within the wounds and enhanced local blood perfusion at the wound site. The ILP100 has progressed to its Phase 2 clinical trial (Identifier: NCT05608187) and is actively enrolling patients with diabetic foot ulcers. This trial spans 26 weeks and includes a long-term follow-up period of 5 years to assess both the safety and biological efficacy of ILP100 in promoting wound healing among subjects.

On the contrary, Zhao et al.^54^ bolstered wound healing mechanisms by applying CXCL12-expressing *Lactococcus lactis* in tandem with yellow light-emitting diodes (LEDs). Prior research has demonstrated that LED light of varying wavelengths exerts distinct effects on skin repair and regeneration. Specifically, yellow light within the range of 570–600 nm can stimulate collagen synthesis, resulting in skin tightening^55^. This synergy expedited wound closure, facilitated tissue remodeling, spurred re-epithelialization and hair follicle regeneration, and mitigated over-inflammation. Additionally, it upregulated pivotal proteins within the Wnt and Notch signaling pathways as well as curtailed inflammatory factors such as interleukin 1 beta (IL-1β) and tumor necrosis factor-alpha (TNF-α). Remarkably, this combined treatment effectively reduced skin pathogens *Ralstonia* and *Acinetobacter*, substantially diminishing the risk of infection. In another instance, Li et al.^56^ devised an inventive approach by combining CXCL12-expressing *L. lactis* with a photosynthetic bacteria, *Synechococcus elongatus* PCC7942, enclosed within a hydrogel matrix. The engineered *L. lactis* feeds on sucrose produced by *S. elongatus* through photosynthesis, creating a synergistic effect that substantially accelerates the wound-healing process. Impressively, this topical hydrogel-encapsulated microbial consortium led to faster wound closure in mice, reducing the wound area ratio to a mere 13.2% by the 14th day, as compared to control treatments.

In addition to inducing chemokines, another strategy employed to expedite wound healing involves augmenting the presence of growth factors, specifically vascular endothelial growth factor (VEGF). This biomolecule is essential in the process of angiogenesis^57^. However, applying VEGF directly has not yielded conclusive benefits in clinical trials^58^. Lu et al.^31^ found that thermosensitive hydrogel containing probiotic *L. lactis* NZ9000 was able to improve the microenvironment of diabetic wounds and promote wound healing by regulating lactic acid levels. They later developed an engineered *L. lactis* carrying a VEGF-encoding gene and embedded it in a heparin-poloxamer hydrogel^59^. This approach enhanced the stability of VEGF in the oxidative environment of chronic wounds and enabled the living system to produce and protect VEGF. As a result, it promoted the growth and movement of endothelial cells, and shifted M1 and M2 macrophages toward an anti-inflammatory phenotype, leading to successful angiogenesis in diabetic wounds^59^.

A prolonged or impaired wound healing may be exacerbated by the biofilm-forming pathogens infection like *S. aureus*, which can be challenging to treat due to their reduced susceptibility to both the immune system and topical antimicrobial agents^60,61^. To address this issue, previous research has explored magnetic hyperthermia as a potential therapeutic modality. Magnetic hyperthermia employed magnetic nanoparticles that absorbed energy from an alternating magnetic field, leading to highly localized heat transmission that inactivated *S. aureus* within cutaneous abscesses in murine models^62^. Building upon this innovative approach, Chen et al.^63^ modified a bacterial system comprising the magneto-ovoid strain MO-1 (closely related to *Magnetococcus* species) which contained magnetosomes and was coated with a polyclonal antibody. In a murine model experiment, this system significantly improved wound healing by promoting the formation of MO-1-*S. aureus* aggregates and eradicates the pathogen by hyperthermia^63^.

### Malignant melanoma therapeutics

Melanoma is a type of skin cancer that is known for its complexity, aggressive nature, high metastasis rate, and frequent relapses. A commonly used method to treat melanoma is phototreatment, where near-infrared (NIR) light and a photosensitizer (PTS) interact to destroy tumor spheroids^64^. However, due to the restricted penetration of NIR light and the low specificity of PTS, melanoma tumors located deep within the skin and phototreatment margins often lead to quick relapse and metastasis. Therefore, Peng et al.^16^ developed a system of transgenic *Escherichia coli* to deliver recombinant human tumor necrosis factor-related apoptosis-inducing ligand (TRAIL) gene. The nanosized outer membrane vesicles (OMVs) produced by the engineered *E. coli* which have been modified with α_v_β_3_ integrin targeting ligand and indocyanine green (ICG) were able to penetrate the stratum corneum and specifically target melanoma cells. When exposed to NIR irritation, these engineered OMVs exhibited photothermal and photodynamic responses against primary melanoma spheroids and activated TRAIL-induced apoptosis in disseminated tumor cells. This resulted in the complete eradication of melanoma^16^.

A different approach utilized to ablate melanoma involved the metabolic engineering of anaerobic oncolytic bacteria, specifically *Clostridium butyricum*^65^. A study conducted nearly five decades ago involving *C. butyricum* revealed its promising oncolytic activity against carcinomas, particularly when used in conjunction with radio-frequency therapy to briefly raise tumor temperatures to the range of 42 to 44 °C^66^. Since then, numerous related studies have been carried out, with recent investigations utilizing a metabolic labeling substrate to enhance its mechanistic action. This substrate was developed by coupling the metabolic substrate of *C. butyricum*, D-alanine, with a photosensitizer known as TPApy, which exhibited aggregation-induced emission. This new metabolic substrate was incorporated into bacterial peptidoglycan to create engineered *C. butyricum*. Once injected into melanoma, this bacterium colonized the hypoxia region and activated the intratumoral immune system thus eradicating the tumor masses. Following this, the peripheral area, which has more oxygen content, caused the bacteria to die off while the photosensitizer on the bacteria exerted a photodynamic effect under light irradiation, further removing any remaining melanoma^65^.

Alternatively, chemotherapy drugs, such as doxorubicin (DOX), can induce immunogenic cell death in tumor cells but have severe long-term side effects due to non-specific drug distribution^67,68^. Natural polysaccharide polymers, like glycogen (GLY), can be used as nanocarriers for drug delivery, providing targeted delivery and controlled drug release, as well as reduced DOX toxicity^69^. On that account, a recent innovative system (GDOX@HSEc) has been developed using engineered *E. coli* to secret heparin sulfatase-1 (HSulf-1) within, and anchoring DOX-linked GLY nanoparticles (GDOX NPs) on the surface^67^. The GDOX@HSEc combination demonstrated a spatiotemporally intratumoral distribution of therapeutic agents. In this context, HSulf-1 was upregulated to restrict angiogenesis and metastasis. Meanwhile, GDOX nanoparticles successfully infiltrated tumor cells, inducing intracellular DNA damage^70^.

Specific constituents of the skin commensals can trigger a robust T cell reaction when they colonize the skin, such as CD8^+^ T cells elicited by *S. epidermidis*^71^. These cells are recognized for their role in fostering skin homeostasis and expediting wound closure^72^. In light of this, Chen et al^73^. engineered *S. epidermidis* NIHLM087 to express various versions of ovalbumin (OVA)-derived MHC class I (OT1) or MHC class II-restricted (OT2) peptides. The most effective approach combined soluble OT1 peptide with cell wall-anchored OT2 peptides, proving highly efficient in triggering immune responses against OVA-expressing B16F10 melanomas in mouse skin. Notably, the absence of either OT1 or OT2 from *S. epidermidis*, the absence of OVA from B16F10 melanomas, or the depletion of CD8^+^ T lymphocytes thwarted the observed anticancer effects, underlining the therapeutic potential of a cellular immune response targeting shared antigens between bacteria and cancer cells^74^.

In previous applications, *Salmonella typhimurium* served as the foundational bacterial chassis for targeting tumors. To enhance safety and ameliorate potential toxicity concerns, a strategic attenuation approach was employed, involving the targeted deletion of specific genes, *purI* and *msbB*, resulting in the creation of a modified strain denoted as VNP20009^75^. While these genetic modifications effectively introduced adenine dependency and mitigated lipopolysaccharide-related toxicity, the clinical utilization of VNP20009 revealed a dose-dependent elevation in proinflammatory cytokines. As a result, this immunological response precipitated adverse effects in patients, encompassing thrombocytopenia, anemia, bacteremia, hyperbilirubinemia, diarrhea, vomiting, and nausea. Presently, efforts are directed towards reducing its toxicity and enhancing tumor localization. In one example, an attenuated *S. typhimurium* expressing recombinant interferon-gamma (IFN-γ) successfully invaded melanoma cells and induced cytotoxicity in melanoma-bearing mice, while showing minimal toxicity to normal cells^76^. A modified strain expressing a radiation-sensitizing microRNA vector encoding the inhibin alpha gene (INHA) was also able to exert cytotoxicity in combination with radiotherapy by enhancing ROS production^77^. Additionally, combining programmed death 1 (PD-1) knockdown with small interfering RNA (siRNA) and pimozide drug therapy effectively suppressed melanoma through caspase 3-mediated apoptosis, as compared to bacteriotherapy alone^78^.

### Status quo in clinical translation of skin eLBPs

Despite substantial progress in eLBP research, only a small fraction of eLBPs for skin therapy has progressed to clinical trials, and they are still in the early stages. Azitra Inc., a pioneering skin eLBP company, is set to enter Phase 1b clinical trials in the first half of 2023 for their leading proprietary drug candidate, ATR-12. This innovative treatment incorporates engineered *S. epidermidis* to produce the serine protease inhibitor Lympho-epithelial Kazal-type related inhibitor (LEKTI) and is aimed at addressing Netherton syndrome (Identifier number pending). In a similar vein, their product ATR-04 is expected to commence Phase 1b clinical trials in the first half of 2024, featuring lyophilized *S. epidermidis* engineered to be auxotrophic to D-alanine as the active ingredient. This product seeks to alleviate the severity of papulopustular rosacea associated with epidermal growth factor receptor inhibitor (EGFRI) therapy (Identifier: NCT04731259). Additionally, Ilya Pharma is currently conducting a Phase 2 clinical trial using *L. reuteri* as a chassis to secrete CXCL12, a short-lived human cytokine, to improve wound healing. The progression of these eLBPs toward clinical trials is a crucial milestone in becoming a viable component of biomedical strategies for addressing human skin diseases.

Crown Aesthetics, a forefront aesthetic manufacturer, has made an impressive addition to its product lineup with the introduction of the BIOJUVE skin biome products, protected by a patent under the Xycrobe® technology (WO2017147507A1). These innovative products primarily consist of engineered *C. acnes* subsp. defendens strain XYCW42 to express human cytokines (such as IL-10, IL-6, IL-7, and IL-8) through an inducible promoter located in front of the *ftsAZ* operon, enabling precise control over bacterial cell division^79^. In their clinical study involving 121 subjects, a specifically designed skincare regimen was rigorously followed including the XYCM42 Ferment Based Serum, Live XYCM42 Gel, and Prebiotic Activator. According to the results, participants demonstrated enhanced skin health with sustained pH balance, optimal transepidermal water loss levels, increased skin moisture, reduced redness, improved skin elasticity, and a noticeable reduction in surface sebum, with particularly pronounced effects in the forehead and nose areas^79^. This significant progress represents a noteworthy milestone for eLBPs within the domain of skin health and beauty products, reinforcing their potential not only in skin repair and therapy but also in advancing towards clinical trials and subsequent commercialization.

### Challenges towards commercialization of eLBPs

To date, the majority of the development of eLBPs has been rooted in probiotics sourced from the gut microbiome, encompassing lactic acid bacteria and commensal strains^80^. This trend can be attributed to the promising potential of eLBPs as biotherapeutics, stemming from their superior safety profile when compared to conventional chemical drugs, especially for extended periods of use^80,81^. eLBPs not only serve as effective therapeutic agents for chronic diseases by colonizing damaged cells but also widen the possibilities for tailored tumor-targeted treatments. The use of eLBPs in treating skin diseases is still in its early stages and must address specific limitations, as detailed by Charbonneau et al.^18^ and Pedrolli et al.^82^, concerning regulatory, application, manufacturing, safety, stability and efficacy. In both the United States and Europe, the development of eLBPs necessitates the establishment of quality by ensuring safety, reliability, robustness, and batch consistency. Nevertheless, regulatory guidance under the purview of the US Food and Drug Administration (FDA) for LBPs remains broad^28^, and there is currently no published directive specifying toxicology requirements tailored to LBPs^18^. Therefore, the development pathway for a specific clinical candidate must involve discussions with the relevant regulatory authorities in the region or country where the product is intended for development and use.

Biocontainment stands as a significant hurdle in the progression of eLBP research. To address this challenge, emerging methods are being explored, with auxotrophies being one notable example. These auxotrophies aim to curtail the proliferation of engineered strains outside their designated environments^9^. For instance, a biocontained *Saccharomyces boulardii* strain, engineered with *THI6* and *BTS1* gene knockouts, exhibited constrained growth in the absence of thiamine concentrations exceeding 1 ng/mL and experienced severe growth impairment at temperatures below 20 °C^83^. Apart from auxotrophies, biocontainment strategies encompass ‘deadman’ and ‘passcode’ kill switches in which the eLBPs are programmed to respond exclusively to precise environmental signals. These switches can suppress the transcription of essential genes in the absence of specific triggers or initiate self-destruction of engineered strains through toxin production, effectively preventing unintended cell proliferation^84^. Furthermore, beyond traditional exo- and endonucleases, CRISPR-associated nucleases (Cas) have been deployed in *E. coli* to design effective kill switches^85^. These kill switch gene circuits leverage Cas3^86^ or Cas9^87^, successfully achieving biocontainment with minimal escape frequencies. A notable advantage of Cas-based kill switches lies in their use of guide RNAs (gRNAs) to selectively target specific DNA sequences or microorganism strains, enabling the precise elimination of the target strain from the microbiome^87,88^. Such innovative biocontainment strategies not only address critical safety concerns but also pave the way for responsible and controlled advancements in the field of eLBPs.

The significance of robust biocontainment in eLBPs research cannot be overstated. Beyond averting unintended consequences and ecological disruptions, it highlights the ethical and safety imperatives essential for the responsible development and utilization of these innovative therapeutic agents. Consequently, the ongoing exploration and enhancement of biocontainment techniques bear profound implications for the progression and acceptance of eLBPs in clinical applications^89^. Apart from clearance or biocontainment, a major future challenge will be addressing issues such as the potential spread of genetically modified LBPs into other bacterial or mammalian cell genomes, how to establish stable colonization of targeted sites, interactions with commensal flora, and tissue targeting. These questions highlight the importance of investigating genetic stability in eLBPs under normal physiological conditions. Although challenging, advancements in biological technologies will enhance the depth and breadth of disease prevention and treatment strategies through the use of newly available bacterial tools and upgraded therapeutic approaches for eLBPs, ultimately alleviating safety concerns.

On a more intricate note, biopharmaceutical manufacturers grapple with a host of challenges when translating LBP concepts (both engineered and non-engineered products) into mass production and commercialization. These challenges span formulation and development, regulatory approval, production and packaging, shipping and storage, patient application, and efficacy. Comprehensive discussions by Vargason and Anselmo et al.^14^ have shed light on the complex landscape confronting biopharmaceutical manufacturers in this pursuit. To make eLBPs viable products that cater to patient needs, it is vital to address these multifaceted challenges. A core necessity lies in foundational research on delivering foreign microbiota to the skin, encompassing queries about formulation design, the impact of common topical formulations on eLBP viability during storage, and the quest for ideal formulations that balance manufacturing practicalities with patient usability. Moreover, analyzing clinical data reveals variations in LBP colonization on the skin, prompting questions about the specific LBP and formulation parameters dictating microbial adherence, competition, growth, and long-term persistence^90^. These complexities necessitate the development of delivery devices and formulations capable of sustaining LBP growth and persistence, even in challenging skin environments, aiming to minimize variability and enhance the overall potential of LBPs in addressing chronic, recurrent, and difficult-to-treat skin conditions, while paving the way for successful mass production and commercialization.

The utilization of eLBPs also brings forth potential risks concerning human safety that demand careful consideration. One critical aspect entails the evaluation of virulence factors within bioengineered strains, necessitating a meticulous screening process to identify genes capable of inducing pathogenicity^91^. Equally vital is the scrutiny of genes responsible for producing enzymes involved in synthesizing toxic or allergenic compounds, or their precursors^92^. The introduction of foreign DNA may lead to the production of novel substances, either as proteins or metabolic byproducts, which can exhibit harmful or allergenic properties. Assessing these newly expressed proteins involves examining amino acid sequences for potential homology and evaluating their stability under various conditions, including heat, processing, and degradation. Despite these measures, precise tests for predicting allergenic responses in humans remain elusive^91^.

Moreover, the safety of eLBPs hinges not only on their composition but also on factors such as the mode of application and the genetic profile of the consumers. Certain vulnerable subpopulations, such as immunocompromised individuals, infants, and the elderly, can exhibit heightened sensitivity, necessitating vigilant post-market surveillance of novel biotherapeutics to avert potential adverse effects. The need for prolonged monitoring after introducing such biotherapeutic products to the market is evident, albeit complicated by technical challenges arising from inconsistent intake. Similarly, in the case of engineered bacteria, vigilant and ongoing monitoring post-release into the market is advisable to preempt delayed adverse effects and guarantee safe consumption^93^. Overall, the safety considerations surrounding the use of eLBPs accentuate the importance of comprehensive risk assessment and vigilant surveillance to safeguard human health. As we explore the potential benefits of these innovative approaches, it is imperative to remain cognizant of the intricate web of factors that can impact their safety profiles and to prioritize ongoing monitoring to ensure the long-term well-being of consumers.

### Prospects for non-commensal bacterial chassis as new eLBPs

Given the progress made in microbial systems biology and synthetic biology^94^, leveraging non-pathogenic skin commensals seems promising for initiating the development of new bacterial chassis dedicated to advancing skin treatment in the field of eLBP. Skin commensal bacterium, *S. epidermidis* is an emerging bioengineered chassis as eLBP in skin disease treatment given the innate anti-staphylococcal activity^36,37^ as well as the successful development of genetic tools for delivery therapeutic proteins such as recombinant filaggrin and LEKTI proteins important for treating AD and microbial dysbiosis^81,95,96^. Nevertheless, genetic manipulation of beneficial strains derived from human-associated microbes presents notable challenges, primarily stemming from the necessity to navigate innate restriction-modification systems. Consequently, these strains are often regarded as genetically intractable when compared to well-established models or known bacterial systems^2,97^.

The discovery of antimicrobial activities of a subset of commensal and non-commensal *Corynebacterium* spp. suggests the possibility of utilizing these bacteria as alternative bacterial chassis for biotherapeutic or biodiagnostic purposes, particularly in targeting skin pathogens like *S. aureus*^98–102^. A recent *Staph*-targeted study has showcased the use of an engineered non-commensal *C. glutamicum* which has been modified to respond to the quorum sensing (QS) molecule known as autoinducing peptide (AIP) produced by *S. aureus*^100^. The expression of accessory regulatory proteins agrAC in tandem with a recombinant red fluorescent protein (RFP) conferred AIP-stimulated protein production in engineered *C. glutamicum* pResponse strain^100^. Given the inherent ability of *C. glutamicum* to hinder the growth of *S. aureus*^100^, this model bacterium can be subjected to additional manipulation to mount a response and combat *Staph* infections, following a strategy akin to that demonstrated by Guan et al.^96^. In this approach, *S. epidermidis* was engineered to produce lysostaphin biomolecules, which effectively inhibit the growth of *S. aureus*^96^.

Compared to *S. epidermidis* and other closely related commensal *Corynebacterium* spp., *C. glutamicum* has been more readily used in the food, feed and biopharmaceutical industry with prior approval from the FDA^103^. Metabolic engineering of AIP-responsive *C. glutamicum* to produce important skin biomolecules such as cobamide^104^ and arginine^105^ will aid in stimulating the production of filaggrin-derived natural moisturizing factors as well as reducing pathogenic *Staph* growth in the skin microbiome. With the expanded availability of CRISPR-Cas genome editing tools^106,107^, unwanted genes that may interfere with the host system can be accurately modified and modulated hence providing a promising means for development of *C. glutamicum* strains as eLBPs. Importantly, the increased interest in the employment of bioengineering and synthetic biology approaches in developing new strains as eLBPs should bring about timely technological development in the race against infectious pathogens following the rules and regulations in countries all over the world.

### Future perspectives

In summary, recent advancements in the development of eLBPs and their clinical trials for tackling skin diseases have ignited significant enthusiasm for delving into the therapeutic potential of these agents. All of these endeavours have been conducted with the highest level of diligence, ensuring the implementation of stringent safety assessment protocols. The substantial body of evidence supporting the efficacy of non-engineered LBPs in maintaining a healthy skin microbiome and their potential as chassis organisms underscores the promising prospects for utilizing eLBPs in skin disease intervention. Nonetheless, it is imperative to acknowledge the multifaceted challenges associated with advancing eLBPs, encompassing issues related to manufacturing scalability, ensuring stability throughout the production process, and the implementation of robust biocontainment strategies. These formidable challenges emphasize the need for comprehensive research and development efforts to effectively address them, facilitating a seamless transition into the clinical phase.

The significant progress achieved through the bioengineering of skin commensals, particularly *S. epidermidis*, and non-commensals like *L. lactis* has established a promising foundation for expanding eLBP development. This pioneering work serves as a blueprint for harnessing other non-pathogenic and non-commensal bacteria, precisely tailored to combat skin pathogens and alleviate inflammatory responses. By leveraging microbial engineering and synthetic biology approaches, this emerging platform holds tremendous potential for revolutionizing the field of skin disease intervention through the development of innovative eLBPs.

### Supplementary information

Supplementary Materials

## References

[1] Mohajeri MH (2018). The role of the microbiome for human health: from basic science to clinical applications. Eur. J. Nutr..

[2] Aggarwal N (2023). Microbiome and human health: current understanding, engineering and enabling technologies. Chem. Rev..

[3] Fyhrquist N (2019). Microbe-host interplay in atopic dermatitis and psoriasis. Nat. Commun..

[4] Chilicka K, Dzieńdziora-Urbińska I, Szyguła R, Asanova B, Nowicka D (2022). Microbiome and probiotics in acne vulgaris—A narrative review. Life.

[5] Tett A (2017). Unexplored diversity and strain-level structure of the skin microbiome associated with psoriasis. npj Biofilms Microbiomes.

[6] Mohsen S, Dickinson JA, Somayaji R (2020). Update on the adverse effects of antimicrobial therapies in community practice. Can. Fam. Phys..

[7] Patangia DV, Anthony Ryan C, Dempsey E, Paul Ross R, Stanton C (2022). Impact of antibiotics on the human microbiome and consequences for host health. Microbiologyopen.

[8] He L (2023). Antibiotic treatment can exacerbate biofilm-associated infection by promoting quorum cheater development. npj Biofilms Microbiomes.

[9] Rutter, J. W., Dekker, L., Owen, K. A. & Barnes, C. P. Microbiome engineering: engineered live biotherapeutic products for treating human disease. *Front. Bioeng. Biotechnol*. **10**, (2022). 10.3389/fbioe.2022.1000873 PMC9523163 36185459

[10] Paquet JC (2021). Entering first-in-human clinical study with a single-strain live biotherapeutic product: Input and feedback gained from the EMA and the FDA. Front. Med..

[11] Heavey MK, Durmusoglu D, Crook N, Anselmo AC (2022). Discovery and delivery strategies for engineered live biotherapeutic products. Trends Biotechnol..

[12] Ağagündüz D (2022). Recent developments in the probiotics as live biotherapeutic products (LBPs) as modulators of gut brain axis related neurological conditions. J. Transl. Med..

[13] Schemczssen-Graeff Z, Pileggi M (2022). Probiotics and live biotherapeutic products aiming at cancer mitigation and patient recover. Front. Genet..

[14] Vargason AM, Anselmo AC (2021). Live biotherapeutic products and probiotics for the skin. Adv. NanoBiomed Res..

[15] Öhnstedt E (2022). Accelerated wound healing in minipigs by on-site production and delivery of CXCL12 by transformed lactic acid bacteria. Pharmaceutics.

[16] Peng LH (2020). Engineering bacterial outer membrane vesicles as transdermal nanoplatforms for photo-TRAIL-programmed therapy against melanoma. Sci. Adv..

[17] Riglar DT, Silver PA (2018). Engineering bacteria for diagnostic and therapeutic applications. Nat. Rev. Microbiol..

[18] Charbonneau MR, Isabella VM, Li N, Kurtz CB (2020). Developing a new class of engineered live bacterial therapeutics to treat human diseases. Nat. Commun..

[19] Omer R (2022). Engineered bacteria-based living materials for biotherapeutic applications. Front. Bioeng. Biotechnol..

[20] Grice EA (2009). Topographical and temporal diversity of the human skin microbiome. Science (80-).

[21] Byrd AL, Belkaid Y, Segre JA (2018). The human skin microbiome. Nat. Rev. Microbiol..

[22] Bouslimani A (2019). The impact of skin care products on skin chemistry and microbiome dynamics. BMC Biol..

[23] Kapono CA (2018). Creating a 3D microbial and chemical snapshot of a human habitat. Sci. Rep..

[24] Murphy B (2022). Alteration of barrier properties, stratum corneum ceramides and microbiome composition in response to lotion application on cosmetic dry skin. Sci. Rep..

[25] Khadka VD (2021). The skin microbiome of patients with atopic dermatitis normalizes gradually during treatment. Front. Cell. Infect. Microbiol..

[26] Olejniczak-Staruch I (2021). Alterations of the skin and gut microbiome in psoriasis and psoriatic arthritis. Int. J. Mol. Sci..

[27] Brandwein M, Steinberg D, Meshner S (2016). Microbial biofilms and the human skin microbiome. npj Biofilms Microbiomes.

[28] FDA. Early clinical trials with live biotherapeutic products: chemistry, manufacturing, and control information. *Guid. Ind*. 1–20 (2016).

[29] Cordaillat-Simmons M, Rouanet A, Pot B (2020). Live biotherapeutic products: the importance of a defined regulatory framework. Exp. Mol. Med..

[30] Martín R, Langella P (2019). Emerging health concepts in the probiotics field: streamlining the definitions. Front. Microbiol..

[31] Fang Z (2020). *Bifidobacteria adolescentis* regulated immune responses and gut microbial composition to alleviate DNFB-induced atopic dermatitis in mice. Eur. J. Nutr..

[32] Umborowati MA (2022). The role of probiotics in the treatment of adult atopic dermatitis: a meta-analysis of randomized controlled trials. J. Heal. Popul. Nutr..

[33] Zhou X (2021). Nicotinamide mononucleotide combined with Lactobacillus fermentum TKSN041 reduces the photoaging damage in murine skin by activating AMPK signaling pathway. Front. Pharmacol..

[34] Lebeer S (2022). Selective targeting of skin pathobionts and inflammation with topically applied lactobacilli. Cell Rep. Med..

[35] Uberoi A (2021). Commensal microbiota regulates skin barrier function and repair via signaling through the aryl hydrocarbon receptor. Cell Host Microbe.

[36] Iwase T (2010). *Staphylococcus epidermidis* Esp inhibits *Staphylococcus aureus* biofilm formation and nasal colonization. Nature.

[37] Pastar I (2020). *Staphylococcus epidermidis* boosts innate immune response by activation of Gamma Delta T cells and induction of Perforin-2 in human skin. Front. Immunol..

[38] Dubin G (2001). Molecular cloning and biochemical characterization of proteases from *Staphylococcus epidermidis*. Biol. Chem..

[39] Marito S, Keshari S, Huang CM (2020). Peg-8 laurate fermentation of *Staphylococcus epidermidis* reduces the required dose of clindamycin against *Cutibacterium acnes*. Int. J. Mol. Sci..

[40] Marito S (2021). Electricity-producing *Staphylococcus epidermidis* counteracts *Cutibacterium acnes*. Sci. Rep..

[41] Yang AJ (2019). A microtube array membrane (MTAM) encapsulated live fermenting *Staphylococcus epidermidis* as a skin probiotic patch against *Cutibacterium acnes*. Int. J. Mol. Sci..

[42] Nakatsuji T (2018). A commensal strain of *Staphylococcus epidermidis* protects against skin neoplasia. Sci. Adv..

[43] Myles IA (2018). First-in-human topical microbiome transplantation with *Roseomonas mucosa* for atopic dermatitis. JCI insight.

[44] Myles IA (2020). Therapeutic responses to Roseomonas mucosa in atopic dermatitis may involve lipid-mediated TNF-related epithelial repair. Sci. Transl. Med..

[45] Callewaert C, Knödlseder N, Karoglan A, Güell M, Paetzold B (2021). Skin microbiome transplantation and manipulation: current state of the art. Comput. Struct. Biotechnol. J..

[46] Perin B, Addetia A, Qin X (2019). Transfer of skin microbiota between two dissimilar autologous microenvironments: a pilot study. PLoS One.

[47] Callewaert C, Lambert J, Van de Wiele T (2017). Towards a bacterial treatment for armpit malodour. Exp. Dermatol..

[48] Kumar P (2022). The cure from within? a review of the microbiome and diet in melanoma. Cancer Metastasis Rev..

[49] Pessemier (2021). Gut–skin axis: current knowledge of the interrelationship between microbial dysbiosis and skin conditions. Microorganisms.

[50] Mashiah J (2022). Clinical efficacy of fecal microbial transplantation treatment in adults with moderate-to-severe atopic dermatitis. Immun. Inflamm. Dis..

[51] Tanoue T (2019). A defined commensal consortium elicits CD8 T cells and anti-cancer immunity. Nature.

[52] Vågesjö E (2018). Accelerated wound healing in mice by on-site production and delivery of CXCL12 by transformed lactic acid bacteria. Proc. Natl Acad. Sci..

[53] Öhnstedt E (2023). Engineered bacteria to accelerate wound healing: an adaptive, randomised, double-blind, placebo-controlled, first-in-human phase 1 trial. eClinicalMedicine.

[54] Zhao X (2021). Combination of an engineered *Lactococcus lactis* expressing CXCL12 with light-emitting diode yellow light as a treatment for scalded skin in mice. Microb. Biotechnol..

[55] Oh PS, Jeong HJ (2019). Therapeutic application of light emitting diode: Photo-oncomic approach. J. Photochem. Photobiol. B Biol..

[56] Li L (2023). Hydrogel-encapsulated engineered microbial consortium as a photoautotrophic “living material” for promoting skin wound healing. ACS Appl. Mater. Interfaces.

[57] Apte RS, Chen DS, Ferrara N (2019). VEGF in signaling and disease: Beyond discovery and development. Cell.

[58] Crawford Y, Ferrara N (2009). VEGF inhibition: Insights from preclinical and clinical studies. Cell Tissue Res..

[59] Lu Y (2021). Engineering bacteria-activated multifunctionalized hydrogel for promoting diabetic wound healing. Adv. Funct. Mater..

[60] Del Giudice P (2020). Skin infections caused by *Staphylococcus aureus*. Acta Derm. Venereol..

[61] Wang ZF (2018). A phage lysin fused to a cell-penetrating peptide kills intracellular methicillin-resistant *Staphylococcus aureus* in keratinocytes and has potential as a treatment for skin infections in mice. Appl. Environ. Microbiol..

[62] Kim MH (2013). Magnetic nanoparticle targeted hyperthermia of cutaneous *Staphylococcus aureus* infection. Ann. Biomed. Eng..

[63] Chen C (2016). Killing of *Staphylococcus aureus* via magnetic hyperthermia mediated by magnetotactic bacteria. Appl. Environ. Microbiol..

[64] Honors CN, Kruger CA, Abrahamse H (2018). Photodynamic therapy for metastatic melanoma treatment: a review. Technol. Cancer Res. Treat..

[65] Shi L (2022). Living bacteria-based immuno-photodynamic therapy: metabolic labeling of *Clostridium butyricum* for eradicating malignant melanoma. Adv. Sci..

[66] Dietzel F, Gericke D, König W (1976). Tumor hyperthermia using high frequency for increase of oncolysis by *Clostridium butyricum* (M 55). Strahlentherapie.

[67] Yang M (2023). Engineered bacteria combined with doxorubicin nanoparticles suppress angiogenesis and metastasis in murine melanoma models. Acta Biomater..

[68] Zhao N, C Woodle M, Mixson AJ (2018). Advances in delivery systems for doxorubicin. J. Nanomed. Nanotechnol..

[69] Yang S (2021). Cancer-activated doxorubicin prodrug nanoparticles induce preferential immune response with minimal doxorubicin-related toxicity. Biomaterials.

[70] Huang, T. et al. Immunogenic cell death effects induced by doxorubicin improved chemo-immunotherapy via restoration of granzyme B activity. *Nano Res*. (2023)

[71] Naik S (2012). Compartmentalized control of skin. Science.

[72] Harrison OJ (2019). Commensal-specific T cell plasticity promotes rapid tissue adaptation to injury. Science (80-).

[73] Chen YE (2023). Engineered skin bacteria induce antitumor T cell responses against melanoma. Science.

[74] Kepp O, Zitvogel L, Kroemer G (2023). Prevention and treatment of cancers by tumor antigen-expressing *Staphylococcus epidermidis*. Oncoimmunology.

[75] Toso JF (2002). Phase I study of the intravenous administration of attenuated Salmonella typhimurium to patients with metastatic melanoma. J. Clin. Oncol..

[76] Yoon W (2017). Application of genetically engineered *Salmonella typhimurium* for interferon-gamma–induced therapy against melanoma. Eur. J. Cancer.

[77] Yoon W, Park Y, Kim S, Park Y, Kim CY (2021). Combined therapy with microRNA-expressing *Salmonella* and irradiation in melanoma. Microorganisms.

[78] Zhao T (2019). PD-1-siRNA delivered by attenuated *Salmonella* enhances the antimelanoma effect of pimozide. Cell Death Dis..

[79] Rhee MS (2023). Characterization of a live *Cutibacterium acnes* subspecies defendens strain XYCM42 and clinical assessment as a topical regimen for general skin health and cosmesis. J. Cosmet. Dermatol..

[80] Börner RA, Kandasamy V, Axelsen AM, Nielsen AT, Bosma EF (2019). Genome editing of lactic acid bacteria: Opportunities for food, feed, pharma and biotech. FEMS Microbiol. Lett..

[81] Dodds D (2020). Controlling the growth of the skin commensal *Staphylococcus epidermidis* using d-alanine auxotrophy. mSphere.

[82] Pedrolli DB (2019). Engineering microbial living therapeutics: the synthetic biology toolbox. Trends Biotechnol..

[83] Hedin KA, Kruse V, Vazquez-Uribe R, Sommer MOA (2023). Biocontainment strategies for in vivo applications of *Saccharomyces boulardii*. Front. Bioeng. Biotechnol..

[84] Chan CTY, Lee JW, Cameron DE, Bashor CJ, Collins JJ (2016). ‘Deadman’ and ‘Passcode’ microbial kill switches for bacterial containment. Nat. Chem. Biol..

[85] Pavão G, Sfalcin I, Bonatto D (2023). Biocontainment techniques and applications for yeast biotechnology. Fermentation.

[86] Caliando BJ, Voigt CA (2015). Targeted DNA degradation using a CRISPR device stably carried in the host genome. Nat. Commun..

[87] Rottinghaus AG, Ferreiro A, Fishbein SRS, Dantas G, Moon TS (2022). Genetically stable CRISPR-based kill switches for engineered microbes. Nat. Commun..

[88] Rottinghaus AG, Vo S, Moon TS (2023). Computational design of CRISPR guide RNAs to enable strain-specific control of microbial consortia. Proc. Natl Acad. Sci..

[89] Liu Y, Feng J, Pan H, Zhang X, Zhang Y (2022). Genetically engineered bacterium: principles, practices, and prospects. Front. Microbiol..

[90] Oh J (2014). Biogeography and individuality shape function in the human skin metagenome. Nature.

[91] Plavec TV, Berlec A (2020). Safety aspects of genetically modified lactic acid bacteria. Microorganisms.

[92] Mathipa MG, Thantsha MS (2017). Probiotic engineering: towards development of robust probiotic strains with enhanced functional properties and for targeted control of enteric pathogens. Gut Pathog..

[93] de Simone C (2019). The unregulated probiotic market. Clin. Gastroenterol. Hepatol..

[94] Leggieri PA (2021). Integrating systems and synthetic biology to understand and engineer microbiomes. Annu. Rev. Biomed. Eng..

[95] Koh LF, Ong RY, Common JE (2022). Skin microbiome of atopic dermatitis. Allergol. Int..

[96] Guan C (2022). Engineering a “detect and destroy” skin probiotic to combat methicillin-resistant *Staphylococcus aureus*. PLoS One.

[97] Costa SK, Donegan NP, Corvaglia AR, François P, Cheung AL (2017). Bypassing the restriction system to improve transformation of *Staphylococcus epidermidis*. J. Bacteriol..

[98] Ramsey MM, Freire MO, Gabrilska RA, Rumbaugh KP, Lemon KP (2016). *Staphylococcus aureus* shifts toward commensalism in response to *Corynebacterium* species. Front. Microbiol..

[99] Menberu MA (2021). *Corynebacterium accolens* has antimicrobial activity against *Staphylococcus aureus* and methicillin-resistant *S. aureus* pathogens isolated from the sinonasal niche of chronic rhinosinusitis patients. Pathogens.

[100] Ruslan US (2023). Development of *Corynebacterium* glutamicum as *Staphylococcal*-targeting chassis via the construction of autoinducing peptide (AIP)-responsive expression system. Sains Malays..

[101] Hardy BL (2019). Corynebacterium pseudodiphtheriticum exploits *Staphylococcus aureus* virulence components in a novel polymicrobial defense strategy. MBio.

[102] Bomar L, Brugger SD, Yost BH, Davies SS, Lemon KP (2016). *Corynebacterium accolens* releases antipneumococcal free fatty acids from human nostril and skin surface triacylglycerols. MBio.

[103] Wolf S (2021). Advances in metabolic engineering of *Corynebacterium glutamicum* to produce high-value active ingredients for food, feed, human health, and well-being. Essays Biochem..

[104] Swaney MH, Sandstrom S, Kalan LR (2022). Cobamide sharing is predicted in the human skin microbiome. mSystems.

[105] Leung MHY (2023). Skin microbiome differentiates into distinct cutotypes with unique metabolic functions upon exposure to polycyclic aromatic hydrocarbons. Microbiome.

[106] Kim GY (2023). Synthetic biology tools for engineering *Corynebacterium glutamicum*. Comput. Struct. Biotechnol. J..

[107] Wang Q (2021). Advances and perspectives for genome editing tools of *Corynebacterium glutamicum*. Front. Microbiol..

