# Supplementary material for: Recent advances in single-cell engineered live biotherapeutic products research for skin repair and disease treatment
Source: NPJ Biofilms Microbiomes. 2023 Dec 8;9:95. doi: 10.1038/s41522-023-00463-8 (PMC10709320; doi:10.1038/s41522-023-00463-8)
Supplement: Supplementary file 1 — Supplementary Materials [file 41522_2023_463_MOESM1_ESM.pdf]

Supplementary Materials

Recent advances in single-cell engineered live biotherapeutic products research for skin repair and disease treatment

Supplementary Table 1. Microorganisms associated with dysbiosis and common skin conditions and diseases.

Indication	Increased abundance	Decreased abundance	Reference
Atopic dermatitis	*Staphylococcus aureus*	*Staphylococcus epidermidis*, *Corynebacterium*, and *Lactobacillus*	[1–3]
Psoriasis	*Finegoldia*, *Corynebacteria*, *Streptococcus*, and *Staphylococcus*	*Cutibacterium*	[2,4–6]
Rosacea	*Rhabdochlamydia*, CF231, *Bifidobacterium*, *Sarcina*, *Ruminococcus* belonging to the phylum of *Chlamydiae*, *Bacteroidetes*, *Actinobacteria*, and *Lentisphaerae*	*Lactobacillus, Megasphaerae, Acidaminococcus, Hemophilus, Roseburia, Clostridium*	[7]
Squamous cell carcinoma	*Staphylococcus aureus*	*Cutibacterium acnes*	[8]
Melanoma	*Fusobacterium and Trueperella*		[9,10]
Acne vulagaris	*Cutibacterium acnes*		[11,12]
Photoaging	*Corynebacterium, Neisseria, Streptococcus, Candida, and Malassezia*	*Cutibacterium*	[13–15]
Diabetic foot ulcer	*Staphylococcus aureus, Bacteroides fragilis, Proteus mirabilis, Pseudomonas aeruginosa, Streptococcus agalactiae and Escherichia coli, Corynebacterium spp., Finegoldia spp. and Anaerococcus spp.*	Not available	[16,17]

Supplementary Table 2. Live biotherapeutic products used in the treatment of skin conditions and diseases.

Species	Indication	Mechanisms/ Description	Application	Study phase	Reference
Roseomonas mucosa	Atopic dermatitis	Improving epithelial barrier function through production of sphingolipids, cholinergic signaling, and flagellin expression	Topical	Clinical Phase I/II (NCT03018275)	[18,19]
	Atopic dermatitis	Skin dressing made up of polyvinyl pyrrolidone,	Topical	Preclinical	[20]

		polyvinyl alcohol and sodium alginate with live *R. mucosa*			
Staphylococcus cohnii	Atopic dermatitis and psoriasis	Suppressing inflammation by inducing anti-inflammatory and glucocorticoid- related genes	Topical	Preclinical	[21]
Staphylococcus epidermidis	Photoaging	Reducing UV-B induced injuries by formation of 4-hydroxynonenal, cyclobutane pyrimidine dimers, and skin lesions	Topical	Preclinical	[22]
	Photoaging	Butyric acid production modulates pro-inflammatory IL-6 cytokine production	Topical	Preclinical	[23]
	Acne lesions	PEG-8 Laurate fermentation inhibits production of pro-inflammatory MIP-2	Intradermal	Preclinical	[24]
	Acne lesions	PEG-8 Laurate fermentation generates electricity and inhibits *C. acnes*	Intradermal	Preclinical	[25]
	Acne lesions	Glycerol fermentation inhibits *C. acnes* and MIP-2 production	Intradermal	Preclinical	[26]
Staphylococcus hominis	Atopic dermatitis Netherton syndrome, psoriasis and rosacea	*S. hominis* killed *S. aureus* and inhibited expression of a toxin *psmα*	Topical	Clinical Phase I (NCT03151148)	[27]
Staphylococcus aureus	MRSA infection	Glycerol fermentation induces short-chain fatty acids and systemic antibody production	Topical	Preclinical	[28]
Spirulina platensis	Cutaneous wounds	Photodynamic destruction of infectious bacteria through ROS production induced by laser irradiation	Topical	Preclinical	[29]
	Cutaneous wounds	PSB hydrogel accelerates collagen deposition and granulation tissue formation	Topical	Preclinical	[30]
Rhodobacter johrii	Cutaneous wounds	Photosynthetic bacteria-incorporated hydrogel for wound dressing	Topical	Preclinical	[30]
Bacillus subtilis	Cutaneous wounds	*B. subtilis* in polyvinyl alcohol microparticles as wound dressing	Topical	Preclinical	[31]
Bacillus circulans	Acne lesions	Flavin-mediated electron transfer alleviate the inflammatory response to treat acne vulgaris	Topical	Preclinical	[32]
Bifidobacteria adolescentis	Atopic dermatitis	*Lactobacillus* growth promotion, Treg differentiation, and Th2 responses suppression	Oral	Preclinical	[33]
Lacticaseibacillus rhamnosus, L. plantarum, and *Lactiplantibacillus pentosus*	Acne lesions	Skin hydration improvement and immunomodulatory effects production	Topical	Clinical (NCT04216160)	[34]

Lactococcus lactis	Diabetic wounds	Production of lactic acid and macrophages M1 to M2 polarization	Topical	Preclinical	[35]
Lactobacillus fermentum	Photoaging	Combination with nicotinamide mononucleotide activates AMPK signaling pathway	Oral	Preclinical	[36]

References

1. Bjerre, R. D. *et al.* Skin dysbiosis in the microbiome in atopic dermatitis is site-specific and involves bacteria, fungus and virus. *BMC Microbiol.* **21**, 1–13 (2021).
2. Fyhrquist, N. *et al.* Microbe-host interplay in atopic dermatitis and psoriasis. *Nat. Commun.* **10**, 1–15 (2019).
3. Khadka, V. D. *et al.* The skin microbiome of patients with atopic dermatitis normalizes gradually during treatment. *Front. Cell. Infect. Microbiol.* **11**, 1–10 (2021).
4. Olejniczak-Staruch, I. *et al.* Alterations of the skin and gut microbiome in psoriasis and psoriatic arthritis. *Int. J. Mol. Sci.* **22**, (2021).
5. Boix-Amorós, A. *et al.* Alterations in the cutaneous microbiome of patients with psoriasis and psoriatic arthritis reveal similarities between non-lesional and lesional skin. *Ann. Rheum. Dis.* ard-2022-223389 (2022) doi:10.1136/ard-2022-223389.
6. Quan, C. *et al.* Psoriatic lesions are characterized by higher bacterial load and imbalance between *Cutibacterium* and *Corynebacterium*. *J. Am. Acad. Dermatol.* **82**, 955–961 (2020).
7. Chen, Y. J., Lee, W. H., Ho, H. J., Tseng, C. H. & Wu, C. Y. An altered fecal microbial profiling in rosacea patients compared to matched controls. *J. Formos. Med. Assoc.* **120**, 256–264 (2021).
8. Voigt, A. Y. *et al.* Skin microbiome variation with cancer progression in human cutaneous squamous cell carcinoma. *J. Invest. Dermatol.* **142**, 2773-2782.e16 (2022).

9. Mrázek, J. *et al.* Melanoma-related changes in skin microbiome. *Folia Microbiol. (Praha).* **64**, 435–442 (2019).

10. Mekadim, C., Skalnikova, H. K., Cizkova, J., Cizkova, V. & Palanova, A. Dysbiosis of skin microbiome and gut microbiome in melanoma progression. *BMC Microbiol.* 1–19 (2022) doi:10.1186/s12866-022-02458-5.

11. McLaughlin, J. *et al. Propionibacterium acnes* and acne vulgaris: New insights from the integration of population genetic, multi-omic, biochemical and host-microbe studies. *Microorganisms* **7**, 128 (2019).

12. van Steensel, M. A. M. Acne in the 21st century. *British Journal of Dermatology* vol. 181 647–648 at https://doi.org/10.1111/bjd.18202 (2019).

13. Shibagaki, N. *et al.* Aging-related changes in the diversity of women's skin microbiomes associated with oral bacteria. *Sci. Rep.* **7**, 10567 (2017).

14. Jugé, R. *et al.* Shift in skin microbiota of Western European women across aging. *J. Appl. Microbiol.* **125**, 907–916 (2018).

15. Li, Z. *et al.* New insights into the skin microbial communities and skin aging. *Front. Microbiol.* **11**, 1–13 (2020).

16. Travis, J. *et al.* The microbiome of diabetic foot ulcers: A comparison of swab and tissue biopsy wound sampling techniques using 16S rRNA gene sequencing. *BMC Microbiol.* **20**, 1–14 (2020).

17. Mudrik-Zohar, H. *et al.* Microbiome characterization of infected diabetic foot ulcers in association with clinical outcomes: Traditional cultures versus molecular sequencing methods. *Front. Cell. Infect. Microbiol.* **12**, 1–13 (2022).

18. Myles, I. A. *et al.* Transplantation of human skin microbiota in models of atopic dermatitis. *JCI Insight* **1**, (2019).

19. Myles, I. A. *et al.* Therapeutic responses to *Roseomonas mucosa* in atopic dermatitis

may involve lipid-mediated TNF-related epithelial repair. *Sci. Transl. Med.* **12**, 1–29 (2020).

20. Liu, X. *et al.* Living symbiotic bacteria-involved skin dressing to combat indigenous pathogens for microbiome-based biotherapy toward atopic dermatitis. *Bioact. Mater.* **21**, 253–266 (2023).
21. Ito, Y. *et al. Staphylococcus cohnii* is a potentially biotherapeutic skin commensal alleviating skin inflammation. *Cell Rep.* **35**, 109052 (2021).
22. Balasubramaniam, A. *et al.* Repurposing INCI-registered compounds as skin prebiotics for probiotic *Staphylococcus epidermidis* against UV-B. *Sci. Rep.* **10**, 1–10 (2020).
23. Keshari, S. *et al.* Butyric acid from probiotic *Staphylococcus epidermidis* in the skin microbiome down-regulates the ultraviolet-induced pro-inflammatory IL-6 cytokine via short-chain fatty acid receptor. *Int. J. Mol. Sci.* **20**, (2019).
24. Marito, S., Keshari, S. & Huang, C. M. Peg-8 laurate fermentation of *Staphylococcus epidermidis* reduces the required dose of clindamycin against *Cutibacterium acnes*. *Int. J. Mol. Sci.* **21**, 1–11 (2020).
25. Marito, S. *et al.* Electricity-producing *Staphylococcus epidermidis* counteracts *Cutibacterium acnes*. *Sci. Rep.* **11**, 1–11 (2021).
26. Yang, A. J. *et al.* A microtube array membrane (MTAM) encapsulated live fermenting *Staphylococcus epidermidis* as a skin probiotic patch against *Cutibacterium acnes*. *Int. J. Mol. Sci.* **20**, (2019).
27. Nakatsuji, T. *et al.* Development of a human skin commensal microbe for bacteriotherapy of atopic dermatitis and use in a phase 1 randomized clinical trial. *Nat. Med.* **27**, 700–709 (2021).
28. Yang, J. J. *et al.* Commensal *Staphylococcus aureus* provokes immunity to protect against skin infection of methicillin-resistant *Staphylococcus aureus*. *Int. J. Mol. Sci.*

19, (2018).

29. Li, W., Wang, S., Zhong, D., Du, Z. & Zhou, M. A bioactive living hydrogel: Photosynthetic bacteria mediated hypoxia elimination and bacteria-killing to promote infected wound healing. *Adv. Ther.* **4**, 1–9 (2021).

30. Zhao, E. *et al.* Engineering a photosynthetic bacteria-incorporated hydrogel for infected wound healing. *Acta Biomater.* **140**, 302–313 (2022).

31. Ben David, N. *et al. Bacillus subtilis* in PVA microparticles for treating open wounds. *ACS Omega* **6**, 13647–13653 (2021).

32. Kao, H.-J., Balasubramaniam, A., Chen, C.-C. & Huang, C.-M. Extracellular electrons transferred from honey probiotic *Bacillus circulans* inhibits inflammatory acne vulgaris. *Sci. Rep.* **12**, 1–8 (2022).

33. Fang, Z. *et al. Bifidobacteria adolescentis* regulated immune responses and gut microbial composition to alleviate DNFB-induced atopic dermatitis in mice. *Eur. J. Nutr.* **59**, 3069–3081 (2020).

34. Lebeer, S. *et al.* Selective targeting of skin pathobionts and inflammation with topically applied *lactobacilli*. *Cell Reports Med.* **3**, 100521 (2022).

35. Lu, Y. F., Deng, J., Wang, J. & Luo, G. X. Effects and mechanism of *Lactococcus lactis* thermo-sensitive hydrogel on the wound healing of full-thickness skin defects in diabetic mice. *Chinese J. Burn.* **36**, 1117–1129 (2020).

36. Zhou, X. *et al.* Nicotinamide mononucleotide combined with *Lactobacillus fermentum* TKSN041 reduces the photoaging damage in murine skin by activating AMPK signaling pathway. *Front. Pharmacol.* **12**, 1–17 (2021).
